# Irregularities of the Dental Arch—Abstract

**Published:** 1898-07

**Authors:** Eugene S. Talbot

**Affiliations:** Fellow of the Chicago Academy of Medicine


### Irregularities of the Dental Arch.—Abstract.

BY EUGENE S. TALBOT, M.D., D.D.S., FELLOW OF THE CHICAGO ACADEMY OF MEDICINE.

Read before the Section on Stomatology, American Medical Association. June 9, 1898.

In studying the etiology of irregularities of the dental arch I shall discuss the merits and demerits of some of the latter theories, including those as to the alleged normal dental arch.

Two misleading classifications of what constitutes normal dental arches have been advanced by able practitioners of dentistry. In the American System of Dentistry* (published in 1887) contains an article entitled "Dental and Facial Types," in which the author after discussing temperaments attempts to show that certain forms of dental arches (which he calls types) are characteristic of different temperaments. Thus "the arch of the bilious temperament from cuspid to cuspid is almost flat, the lines backward from these points slightly diverging in an almost straight line. The dome of the mouth is high and almost square. The sanguinous arch resembles a horseshoe in shape. The dome of the mouth is high and semi-circular. The arch of the nervous temperament presents a strong contrast to either of the two preceding, and is sometimes spoken of as gothic from its pointed character. From the central incisors, which often overlap for want of space, the line of the remaining teeth continuing backward with a slight curve, the greatest prominence being between the cuspids and first bicuspids. The roof of the mouth partakes of the same curve and angle as the arch. The lymphatic arch is almost semi-circular in its outline and somewhat resembles the sanguinous temperament. The dome or roof of the mouth is flat and low."

In "The American Text-book of Operative Dentistry," page 18, the author of the chapter on "The Dental Arch" remarks: "The 'Square Arch' (which resembles Ivy's arch of the bilious)

*Vol. 2. Page 1030.

is found usually in persons of strong, osseous organization, of Scotch or Irish descent of Gaelic extraction. The 'round square' (which resembles Ivy's sanguinous) is the medium arch, and is the form usually met with in ordinary, well-developed, robust Americans. The 'Round Arch' is the circular or horse-shoe. This arch is quite characteristic in some races as the brachycephalic, South German. The round V, which resembles Ivy's nervous arch, is constricted in front, or narrow, so that the incisors mark a small curve whose apex is the center. It is the arch of beauty, and that is most manifest in women of the Latin races."

When the causes which produce the changes in outline and deformities are fully understood, the absurdity of the theories from the standpoint of late researches becomes apparent. Since the dental arch cannot be outlined until all the permanent teeth are in position, ("as nothing can act except it exists,") and as these deformities are never observed in connection with the first set of teeth, in order to study the normal or deformed arch the person must have reached the age of from six to twelve years. It is self-evident, then, that the influences which bring about changes in outline must exert themselves at that period, and not earlier. In order, therefore, to understand the normal as well as the abnormal dental arch, the human face and jaws must be studied.

The evolution of the human face and jaws is rather complicated, since their development is the result of two forms of evolution. 1st. The evolution of the face and jaws from the embryo to maturity. 2d. The evolution of the face and jaws from the anthropoid ape to that of the present man.

The human face is modified backward from the vertebrate type. It is an additional illustration of the degeneracy of a series of related structures for the benefit of the organism as a whole. The progress of development of the face in the vertebrates is checked in man. First, as Minot* remarks, because the upright position renders it unnecessary to bend the head, as in quadrupeds. Second, because the enormous cerebral develop-

*Human Embryology, page 468.

ment has rendered an enlargement of the brain cavity necessary. This has taken place by extending the cavity over the nose region, as well as by enlarging the whole skull. Third, because the development of the face is arrested at an embryonic stage; the production of a long snout being really an advance in development, which does not take place in man.

The human face at birth is so near that of the monkey's that, if only the heads of both were exposed to view, it would be difficult for a casual observer to distinguish one from the other. Cope* has made the following classification of the head and face for comparative study:

"The relative size of the cerebral to the facial regions; the prominence of the forehead; the prominence of the superciliary regions; the prominence of the alveolar borders; the prominence and width of the chin; the relation to width of skull; the prominence of the malar bones; the form of the nose; the relative size of the mouth and lips."

"At birth in the infant ape, the facial region of the skull is smaller than in the adult; the forehead is more prominent; the superciliary ridges are more prominent; the edges of the jaws are more prominent; the chin is less, while the cheek bones are more prominent; the nose is without a bridge, and has short and flat cartilages; the face is flattened; the orbits are smaller and closer together; the mouth is small and the lips are thin.'

"In the typical infant, as he begins to develop, the cerebral part of the skull more than in the adult predominates over the facial; the superciliary ridges are not developed; the alveolar borders are not prominent; the malar bones are not prominent; the nose is without a bridge, and the cartilages are flat and generally short; the eyes are larger." In this last particular the human infant resembles the lemurs, and thus retains an embryonic tendency. In some degenerates this tendency remains unchecked, and the result is unusually large orbits. In other instances the human fœtus passes through this lemurian stage to reach and even exceed the anthropoid in smallness and closeness together.

*American Naturalist, 1883.

In a paper read before the Odontographic Society of Chicago and the First District Society of New York, and just published* entitled "Degeneracy in its Relations to Deformities of the Jaws and Irregularities of the Teeth," I showed how evolution of the face progressed from the ape to man until it presented a perpendicular line, and that recession of the jaws was still going on. In the evolution of the jaws from birth to maturity there is constant enlargement of the alveolar process brought about by the crowns of the teeth and broadening of the rami by the development of the skull, which is claimed by some writers to stamp the type of jaw. Tomes† has shown, after repeated experiments, that the inner surfaces of the fœtal jaws remain the same throughout life; that the development and growth is almost entirely upon the outer plate of bone, anteriorly and laterally, and that the jaw grows posteriorly for the purpose of containing the molar teeth. It is this growth that is checked by the other form of evolution which characterizes the race. It is this growth that is checked in development at some period in the evolution of the face and jaws from the fœtus to maturity, the result of degeneracy. Having explained the two forms of evolution which take place in the face and jaws, it is fitting at this point to return to the discussion of the type of jaws.

When man was more of an animal than he is to-day, in other words, when his jaws protruded beyond the perpendicular line, it was possible in brachycephalic and dolichocephalic skulls, to show these distinct forms of dental arches, as represented by Ivy. This, however, could not be relied upon, as I have shown‡ that it is possible in one family, say nothing of nationalities, to change from a complete dolichocephalic head to a most perfect brachycephalic, and *vice versa*, in four generations. Granting that one parent possessed a brachycephalic and the other a dolichocephalic head, what would be the shape of the jaws in the two intermediate generations? These facts are antagonistic to this theory, since the temperaments change more readily and

*Chicago Dental Review, April, 1898.

†Dental Surgery.

‡Talbot, The Etiology of Osseous Deformities of the Head, Face, Jaws and Teeth.

since the jaws are affected particularly by constitutional and local effects. Again, I have also found that the tendency of all nationalities is toward the mesocephalic type of head, and is strongly illustrated in the negro race which is supposed to be a long-headed or dolichocephalic race. In an examination of over 3,000 negroes in Chicago I found only six that were long-headed, and four of these bordered upon the extreme mesocephalic type.

It is easy to see why temperament has very little to do with the shape of the dental arch. Two individuals are married, one a nervous, the other a lymphatic, bilious or sanguinous temperament; all having different jaws, the offspring inherits the jaws of one, teeth of the other, and the temperament of the child is changed. The local condition is such that the shape of the jaw may change the character of the arch entirely. One child may possess a broad dental arch, but very short, another a very narrow and long dental arch. Hence, classifying the dental arch and vault by temperament is wholly out of the question.

Likewise, the theory of placing fixed types of jaws with different nationalities is utterly impossible, since all forms of dental arches are observed in each nationality.

Some of the primitive races, like the negro, Chinese, the Latin races, etc., retain the excessive jaws, while the Anglo-Saxons, Scandinavians and Gaelic races in most cases have passed far within the perpendicular line. It is possible for the arrested jaw to be found among the negro, Chinese and Latin races and large protruding jaws among the Anglo-Saxons, Scandinavians and Gaelic races. These would be atavistic on the one hand and degenerate on the other.

It remains now to explain how irregularities of the dental arch are produced and also why in the present people (bilious, lymphatic and sanguinary), dental arches cannot be fixed and confined to distinct nationalities. It is not until the evolution of the face has assumed the perpendicular line that it becomes of interest to practitioners of dentistry. The teeth do not change (in size) to correspond to the size of the jaws, therefore, at this period, the jaws are as small as they can possibly be to contain them normally.

Some years ago* I divided irregularities of the dental arch into constitutional and local. *Constitutional* because arrest and excessive development of the maxillary bones may be caused by inherited predisposition, but more frequently by constitutional diseases at any period between birth and the sixth year, resulting in an unstable nervous system. These excessive and arrested jaws may become inherited in succeeding generations. I mention the sixth year because these deformities are never observed in connection with the temporary set of teeth, but invariably after that period. Owing to this unstable condition of the jaws, there may be a lateral arrest of development with a protrusion of the jaws beyond the perpendicular line. In such cases there may be V and saddle arches, and the face will assume the hatchet shape. *Local* because irregularities of the teeth may be the result of undue influence of adjoining or occluding teeth. A local condition which might easily be taken for a constitutional cause is where the first permanent molar from any cause may move forward, thus diminishing the space required for the anterior teeth.

In the constitutional variety of irregularities of the teeth arrest of development of the jaws invariably takes place. The jaws being too small to contain all the teeth, nature is trying to overcome this difficulty by dropping the third molars and the lateral incisors, hence almost invariably arrest of development of the jaws exists when these teeth are missing. It not infrequently happens that, although the third molars may be missing, there is not sufficient room for the anterior teeth to develop normally. Given a jaw too small for the long diameter of the teeth, a break must take place in the dental arch.

After eight years of constant study watching the development of jaw deformities and making artificially every movement of the teeth which produces them, I showed in a series of papers† that irregularities of the teeth were merely a mechanical arrangement depending entirely upon the jaw on the one hand, the teeth on the other, and that mouth-breathing, thumb-sucking

*Dental Cosmos, October, 1889.

†Papers on the Etiology of Constitutional Irregularities of the Teeth. Dental Cosmos, 1888–1889.

and other external conditions (which up to that time were supposed to be the cause) had nothing to do with them.

Two typical breaks take place, each depending entirely upon the relation of the cuspid tooth to the other teeth. If the cuspid come into position after the bicuspids have erupted, the break is in the anterior part of the mouth, producing the V variety, which is atavistic, resembling the reptilian type. If the cuspid erupts before the bicuspids, the break is at the sides forming the saddle arch, also atavistic, resembling the carnivarian type. Modifications of these types are the result of local arrangement of the teeth as they erupt. One of the most marked examples of this is where the temporaries are retained too long in the jaw, the roots retaining the crowns of the bicuspids in a smaller circle. When the temporary teeth are removed, the crowns of the bicuspids are deflected inward, from want of room, by the adjoining teeth. The cause and manner of formation of the local variety of irregularities have been described so often in my papers and books that it seems unnecessary to enter into their discussion at this time.

It will be seen, therefore, that a race or individual, regardless of temperament, whose face and jaw (in the order of evolution) have become arrested at any point within the perpendicular line (depending upon local and mechanical construction of the dental arch) cannot possess fixed dental types of jaws and teeth.

